# Clinicopathological and prognostic value of calcification morphology descriptors in ductal carcinoma in situ of the breast: a systematic review and meta-analysis

**DOI:** 10.1186/s13244-023-01529-z

**Published:** 2023-12-05

**Authors:** Merle M. van Leeuwen, Shannon Doyle, Alexandra W. van den Belt–Dusebout, Stevie van der Mierden, Claudette E. Loo, Ritse M. Mann, Jonas Teuwen, Jelle Wesseling

**Affiliations:** 1 Division of Molecular Pathology, Netherlands Cancer Institute - Antoni Van Leeuwenhoek, Amsterdam, the Netherlands; 2 Division of Radiation Oncology, Netherlands Cancer Institute - Antoni Van Leeuwenhoek, Amsterdam, the Netherlands; 3 Scientific Information Services, Netherlands Cancer Institute - Antoni Van Leeuwenhoek, Amsterdam, the Netherlands; 4 Department of Radiology, Netherlands Cancer Institute - Antoni Van Leeuwenhoek, Amsterdam, the Netherlands; 5 Department of Medical Imaging, Radboud University Nijmegen, Nijmegen, the Netherlands; 6 Department of Pathology, Netherlands Cancer Institute - Antoni van Leeuwenhoek, Amsterdam, the Netherlands; 7 Department of Pathology, Leiden University Medical Center, Leiden, the Netherlands

**Keywords:** Ductal carcinoma in situ, Calcification morphology descriptors, Imaging biomarker, Mammography

## Abstract

**Background:**

Calcifications on mammography can be indicative of breast cancer, but the prognostic value of their appearance remains unclear. This systematic review and meta-analysis aimed to evaluate the association between mammographic calcification morphology descriptors (CMDs) and clinicopathological factors.

**Methods:**

A comprehensive literature search in Medline via Ovid, Embase.com, and Web of Science was conducted for articles published between 2000 and January 2022 that assessed the relationship between CMDs and clinicopathological factors, excluding case reports and review articles. The risk of bias and overall quality of evidence were evaluated using the QUIPS tool and GRADE. A random-effects model was used to synthesize the extracted data. This systematic review is reported according to the Preferred Reporting Items for Systematic reviews and Meta-Analyses (PRISMA).

**Results:**

Among the 4715 articles reviewed, 29 met the inclusion criteria, reporting on 17 different clinicopathological factors in relation to CMDs. Heterogeneity between studies was present and the overall risk of bias was high, primarily due to small, inadequately described study populations. Meta-analysis demonstrated significant associations between fine linear calcifications and high-grade DCIS [pooled odds ratio (pOR), 4.92; 95% confidence interval (CI), 2.64–9.17], (comedo)necrosis (pOR, 3.46; 95% CI, 1.29–9.30), (micro)invasion (pOR, 1.53; 95% CI, 1.03–2.27), and a negative association with estrogen receptor positivity (pOR, 0.33; 95% CI, 0.12–0.89).

**Conclusions:**

CMDs detected on mammography have prognostic value, but there is a high level of bias and variability between current studies. In order for CMDs to achieve clinical utility, standardization in reporting of CMDs is necessary.

**Critical relevance statement:**

Mammographic calcification morphology descriptors (CMDs) have prognostic value, but in order for CMDs to achieve clinical utility, standardization in reporting of CMDs is necessary.

**Systematic review registration:**

CRD42022341599

**Key points:**

• Mammographic calcifications can be indicative of breast cancer.

• The prognostic value of mammographic calcifications is still unclear.

• Specific mammographic calcification morphologies are related to lesion aggressiveness.

• Variability between studies necessitates standardization in calcification evaluation to achieve clinical utility.

**Graphical Abstract:**

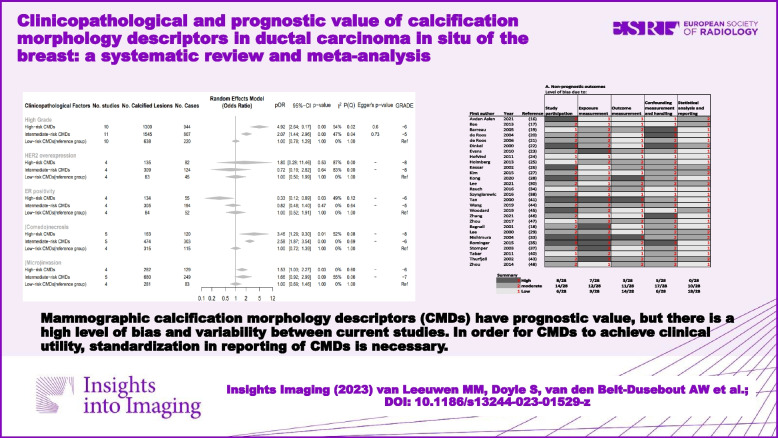

**Supplementary Information:**

The online version contains supplementary material available at 10.1186/s13244-023-01529-z.

## Introduction

Screening mammograms frequently reveal the presence of calcifications, a majority of which are associated with benign changes in breast tissue. However, a minority of these calcifications may be linked to invasive breast cancer (IBC) or its non-obligatory precursor, ductal carcinoma in situ (DCIS) [1].

The introduction of systematic mammographic screening [1–3] has significantly increased DCIS detection, as up to 90% of DCIS lesions are detected due to calcifications on mammography [4]. Nevertheless, the anticipated decrease in mortality due to increased DCIS treatment has not been as significant as expected [1], supporting the corpus of evidence that many DCIS lesions would remain harmless if left untreated [5]. Currently, it is not possible to differentiate accurately between calcifications associated with DCIS that will progress to IBC, i.e., high-risk DCIS, and those that remain indolent, i.e., low-risk DCIS. Consequently, all DCIS cases receive treatment, resulting in overtreatment of low-risk DCIS [6–10].

Given the heterogeneity of DCIS in terms of morphology, biology, genetics, and outcome [11], mammographic calcification patterns and distributions may reflect the disease’s heterogeneity.

The American College of Radiology’s Breast Imaging-Reporting and Data System (BI-RADS) standardizes these patterns, classifying calcifications with a suspicious morphology into four categories, (1) amorphous, (2) coarse heterogeneous, (3) fine pleomorphic, and (4) fine linear or fine linear branching [12]. It is noteworthy that not all mammographically observed calcifications are related to DCIS or invasive breast cancer. Amorphous calcifications are only associated with malignancy in about 20% of cases, whereas the positive predictive value of fine linear calcifications is higher (70–80%) [13, 14]. In DCIS, calcifications are most commonly linear, linear branching, and fine pleomorphic, in a linear distribution [15].

Investigating the association between calcification morphology descriptors (CMDs) and specific clinicopathological factors of DCIS lesions, such as grade, receptor-based surrogate subtypes based on hormone receptors and HER2, and the risk of local DCIS or IBC recurrence could provide clinicians with valuable insights into the likelihood of DCIS progression and lesion aggressiveness. Accurately determined CMDs may enable clinicians to make better-informed decisions regarding the necessity of further diagnostic procedures, such as biopsies. However, despite numerous studies, the prognostic value of mammographic calcification descriptors remains unclear [16–48].

To address this knowledge gap, we conducted a systematic review and meta-analysis to assess the association between mammographic CMDs and clinicopathological factors in women with DCIS, while evaluating the overall quality of evidence and identifying sources of bias.

## Materials and methods

This systematic review is reported according to the Preferred Reporting Items for Systematic reviews and Meta-Analyses (PRISMA) [49]. The study protocol was registered under study ID CRD42022341599 in PROSPERO [50], an international prospective register of systematic reviews.

A comprehensive literature search was performed in Medline via Ovid, Embase.com, and Web of Science Core collection (SCI-expanded, SSCI, A&HCI, ESCI) according to Bramer et al. [51]. Non-peer-reviewed sources such a Google and other grey literature sources were excluded from the search. The literature search focused on English-language articles published from 2000 (when BI-RADS was implemented in the Netherlands) and was conducted on January 25, 2022. Schematically, the search is as follows: (calcinosis AND (mammography or BI-RADS)) AND (DCIS OR breast cancer). Both thesaurus terms (in Medline via Ovid and Embase.com) and free text terms were used if applicable. Conference abstracts were excluded based on the publication type metadata. The search strategy did not employ any additional filters or draw from previous searches. The scope and syntax of the search were verified by a second information specialist. Supplementary Table S1 provides a comprehensive description of the search strategy.

The search results were imported into EndNote 20 [52] to remove duplicate records and retrieve articles. Duplicates were removed using the Bramer method, a specialized technique designed to increase accuracy when compared to automatic deduplication by a reference management system [53]. Initial screening of titles and abstracts was conducted by both M.M.L. and S.D. using the Rayyan app [54]. Studies were considered eligible if they detailed the mammographic CMDs related to clinicopathological factors such as grade, receptor-based surrogate subtypes based on hormone receptors and HER2, and risk of local DCIS or IBC recurrence. M.M.L. and S.D. independently screened the remaining full-text articles for inclusion, resolving discrepancies through group discussion with team members. Exclusion criteria were documented as follows: (1) non-original data (e.g., reviews, editorials, and guidelines), (2) non-English articles, (3) preclinical studies (e.g., animal or in vitro studies), (4) case reports and very small studies (i.e., studies including less than 20 DCIS patients with calcifications), (5) arterial calcifications, (6) other breast imaging techniques than mammography or experimental breast imaging modalities (e.g., ultrasound), and (7) calcification morphology not described. Subsequently, the reference lists of included articles were examined for any relevant articles not identified by the search. Finally, articles with overlapping patient data were excluded, retaining only the largest series.

No other sources were searched, and no other methods were used.

### Data extraction and definitions

After the selection process, a custom form was used to extract several study characteristics of the included articles. These characteristics included study details (reference, country, study design), patient and lesion characteristics (setting of recruitment, single- or multi-center, follow-up time in years if applicable, number of DCIS or IBC lesions presenting as calcifications (only), age in years, histopathological size of the lesion), outcome measurement details (type of assay), exposure measurement details (imaging system, method of detection, number of (blinded) readers, calcification classification system), and necessary information for quality assessment.

The numerical results were documented by cross-tabulating each CMD’s absolute numbers concerning clinicopathological factors.

Authors were not contacted for missing data. Missing information was noted as “not specified” or “not available”.

### Quality assessment

The Quality in Prognostic Studies (QUIPS) tool [55] was used to assess the risk of bias in prognostic and non-prognostic outcomes, covering six domains: study participation, study attrition, exposure measurement, outcome measurement, study confounding, and statistical analysis and reporting. Each domain was evaluated using three to six related questions. Supplementary Table S2 provides more details on the assessment tool. Each study was given a low, moderate, or high risk of bias for each study, with low risk marked as one and high risk as three. The study attrition domain was only rated for prognostic studies that involved follow-up. Some studies reported both prognostic (e.g., recurrence and progression to IBC) and non-prognostic outcomes, with the QUIPS tool applied separately for these outcomes.

To ensure consistency in the interpretation of the QUIPS criteria, the first five papers’ results were compared between the two reviewers. Then, M.M.L. and S.D. independently performed quality assessment of the included studies. A low-high discrepancy was defined as a difference in risk of bias rating between the two reviewers for a specific domain. When the reviewers did not reach a consensus in case of a low-high discrepancy, they sought the help of a third reviewer (A.W.B.D.), to reach a consensus decision.

The average risk of bias per QUIPS domain was compared using a *t*-test for studies published between 2000 and 2010 and between 2010 and 2022, with high risk of bias scored as three, medium risk as two, and low risk as one.

### Certainty of evidence

The certainty of evidence (CoE) for studies included in the meta-analysis was assessed using a modified GRADE (Grading of Recommendations, Assessment, Development, and Evaluations) instrument [56]. Since all included studies were observational, the initial overall quality of evidence graded as low with a score of 0. Subsequently, the overall quality rating was adjusted based on the following domains: risk of bias, inconsistency, indirectness, imprecision, and publication bias. A domain rated as having a “moderate to low quality of evidence” was downgraded by one point, while a domain rated as having a “very low quality of evidence” was downgraded by two points. Supplementary Table S3 contains the reasons for downgrading.

The risk of bias was determined using the average QUIPS tool score. Inconsistency (heterogeneity) was determined through *I*^2^ values and the Cochran’s *Q*-test’s *p*-value (*P*(*Q*)). *I*^2^ values above 50% and a significant *Q*-test indicated high heterogeneity between the studies. Indirectness was assessed by examining differences between studies in population characteristics, exposure, and outcome measurements. Imprecision was evaluated based on point estimates spread, 95% confidence intervals size, and the overlap of confidence intervals. Publication bias was assessed using the Egger’s test. To account for the test’s lack of power without a representative number of studies [57], it was applied only to outcomes with at least 10 studies. The GRADE score was downgraded by one point for studies whose publication bias could not be assessed.

### Data synthesis

The total number of studies assessing the same clinicopathological factor in relation to CMDs was cross-tabulated along with the number of studies finding a significant association with CMDs.

The associations between CMDs and clinicopathological factors were synthesized in pooled odds ratios (pORs) after grouping CMDs into three risk categories. CMDs were categorized into a low-risk, intermediate-risk, and high-risk group for meta-analysis since not all studies used BI-RADS descriptors or analyzed different CMDs separately. The low-risk group included calcifications with punctate or amorphous morphology and served as the reference group. The intermediate-risk group consisted of calcifications with a coarse heterogeneous or (fine) pleomorphic morphology, while the high risk-group included fine linear calcifications based on the difference in positive predictive value for the presence of DCIS. To categorize CMDs into risk groups, non-BI-RADS descriptors were aligned with BI-RADS descriptors (Supplementary Table S4) based on similar descriptions (e.g., “linear” and “casting-type” as both describe calcifications arranged in a line).

Random-effects models were employed to calculate the pORs for CMDs and clinicopathological factors, allowing for heterogeneity between studies. The Mantel-Haenszel method was used to combine binary effect estimates (ORs) across studies. The Paule-Mandel estimator was used to model between-study variance (*tau*^*2*^) in calculating the pORs, as this estimator is suitable for studies with small sample sizes and binary outcomes. A forest plot was used to visually represent the pOR for each factor and risk group.

Studies were excluded from the meta-analysis if they did not report effect sizes or used categories (e.g., effect size measures, follow-up period, definitions or methods of measurement for exposure or outcome, adjustment factors, and analytical methods) that were not comparable with the other studies assessing that specific clinicopathological factor.

Subgroup analyses were not feasible due to the limited number of studies per clinicopathological factor and insufficient information on relevant subgroups (e.g., method of detection, calcification classification systems, and number of readers).

All analyses were performed with R version 4.2.2 (R Foundation for Statistical Computing, Vienna, Austria) using the meta R package (version 6.1). Pooled estimates were reported in combination with 95% confidence intervals (CI), and two-sided *p*-values of < 0.05 were used to determine statistical significance.

## Results

Through an extensive search that included reference cross-checking of relevant articles, a total of 4715 unique articles were collected. An initial screening, based only on studies’ titles, led to the exclusion of 3946 articles. A following evaluation of the remaining 769 articles, based on their abstract, led to the removal of 666 articles. Among these, about 44% were excluded because they were not in English, presented non-original data, or were classified as case reports. Further analysis of the remaining 103 articles using full-text assessment (Supplementary Table S5) resulted in the exclusion of another 74 articles. The main reason of exclusion at this stage was the lack of an adequate description of calcification morphology.

Ultimately, 29 studies met the strict inclusion criteria, which covered CMDs and associated clinicopathological factors in patients diagnosed with DCIS. The results section is further organized in three sections: (i) study characteristics, presenting an overview of the included studies and patient populations; (ii) results of synthesis, consolidating the extracted outcomes from the included studies; and (iii) assessment of bias using the QUIPS tool, evaluating the risk of bias across the selected studies.

**Fig. 1:**
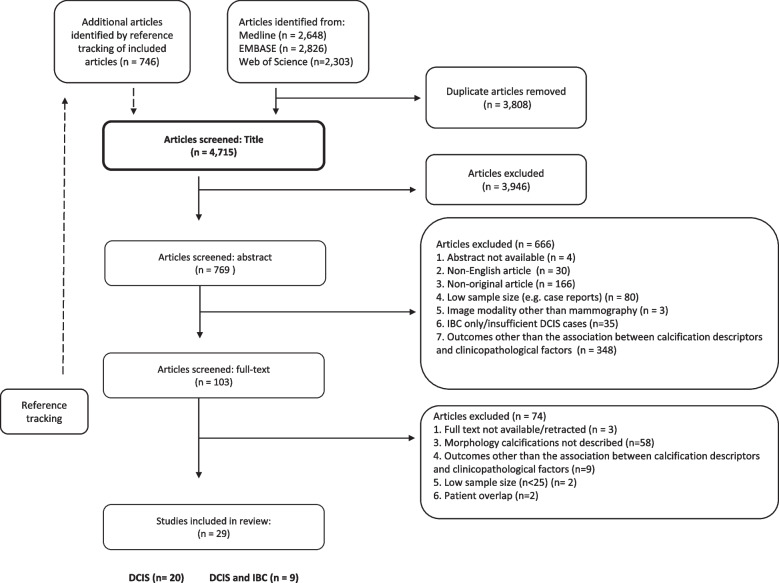
Overview of the Medline, EMBASE, and Web of Science literature search and selection process of eligible articles. The searches were performed on January 25, 2022. Note that 4713 articles were identified, of which 29 met our inclusion criteria. Abbreviations: DCIS, ductal carcinoma in situ; IBC, invasive breast cancer

Figure 1 outlines the approach used for the systematic literature search and subsequent study selection process.

### Study characteristics

In all 29 studies, the data was collected retrospectively from hospital registries, national registries, or clinical trials. Cohort (*n* = 27), case–control (*n* = 1), and a case-cohort within a random control trial (*n* = 1) designs were used. Twenty were single-center studies, and 9 involved multiple centers. While 20 studies exclusively studied DCIS, nine studies included patients with both DCIS and IBC. The number of DCIS patients with calcifications per study ranged from 32 to 1783. Fifteen studies described calcification morphology according to the BI-RADS system, while the remaining 14 studies used non-BI-RADS descriptors. In ten studies, the lesions were described as only screen-detected, while in another ten studies, they were reported to be both screen-detected and non-screen-detected. The remaining studies did not specify the method of lesion detection. Thirteen studies specified using mammograms with calcifications only, without other mammographic abnormalities.

Table 1 shows the characteristics of the selected studies between 2000 and 2022.

**Table 1:** Characteristics of studies reporting on mammographic morphology of calcifications associated with clinicopathological factors

**Study characteristics**		**Patients**							**Assessment clinicopathological factors**	
									**Molecular subtype**	
*Ref., country, recruitment period*	*(Observational) study design*	*Setting of recruitment*	*Single- or multi-center*	*Follow-up (years)*	*No. of DCIS patients with calcifications used in analysis*	*No. of lesions presenting as calcifications only*	*Mean age in years (range/SD) of study population*	*Mean histologic size of DCIS in cm (range/SD)*	*Assay receptor expression*	*Cut-off*
Avdan Aslan et al. 2021 [16], Turkey, 2007–2013	Cohort	University hospital	Single	N/A	66	NS	53 (24–73)^a^	NS (0.4–13.4)	IHC NOS	ER/PR: ≥ 10% nuclear staining Her2: IHC 3+
Bae et al. 2013 [17], South Korea, 2006–2011	Cohort	University hospital	Single	N/A	101	101	36 (27–83)	3.7 (0.5–12.3)^a^	IHC NOS; FISH PathVysion	ER/PR: ≥ 10% nuclear staining Her2: IHC 3+ or (IHC 2+ and FISH+)
Bagnall et al. 2001 [18], England, 1994–1999	Cohort	City hospital	Single	N/A	DCIS: 78 DCIS + IBC: 38	116	58 (40–80)	NS	N/A	N/A
Barreau et al. 2005 [19], France, 1980–1999	Cohort	Regional cancer center	Single	N/A	686	NS	52.4 (19–88)	NS	N/A	N/A
de Roos et al. 2006 [21], The Netherlands, 1991–2003	Cohort	Hospital	Single	N/A	77	NS	58.8 (NS)^a^	2.6^a^	N/A	N/A
de Roos et al. 2004 [20], the Netherlands, 1986–2000	Cohort	Hospital	Single	N/A	87	70	59 (NS)	3.6 (NS)	N/A	N/A
Dinkel et al. 2000 [22], Germany, 1993–1998	Cohort	University hospital	Single	N/A	47	NS	53.4 (26.5–77.3)	1.28 (0.4–4)	N/A	N/A
Evans et al. 2010 [23], UK, NS	Cohort	Nationwide cohort	Multi	N/A	1783	NS	NS (50–69)	NS (NS)	N/A	N/A
Hofvind et al. 2011 [24], Norway, 1995–2007	Cohort	Nationwide cohort	Multi	N/A	202	NS	58.3 (49.3–71.2)	NS (NS)	N/A	N/A
Holmberg et al. 2013 [25], Sweden, 1987–1999	Case-cohort study within a randomized trial	Nationwide cohort	Multi	8	162	NS	Subcohort: 56.7 Cases outside: 55.4	NS (NS)	N/A	N/A
Kessar et al. 2002 [26], England, 1989–1996	Cohort	Hospital	Single	N/A	102	58	58 (25–89)	NS (NS)	N/A	N/A
Kim et al. 2015 [27], South Korea, 2007–2013	Cohort	University hospital	Single	N/A	77	66	53.8 (26–74)	Non-calcified: 2.2 (2.1) Calcified 2.8 (2.2)	IHC NOS; SISH NOS	ER/PR: Allred score ≥ 3 Her2: IHC 3+ or (IHC 2+ and SISH+)
Kong et al. 2020 [28], China, January 2018–December 2018	Cohort	University hospital	Single	N/A	79	NS	48 (40–57)	NS	N/A	N/A
Lee et al. 2000 [29], US, 1992–1999	Cohort	Yale University School of Medicine	Single	N/A	DCIS: 42 DCIS + IBC: 17	NS	59 (36–86)	NS	N/A	N/A
Lee et al. 2021 [30], South Korea, 2009–2019	Cohort	University hospital	Single	N/A	83	NS	52.3 (31–83)	NS (NS)	IHC NS; dual ISH (Ventana medical systems)	NS
Lilleborge et al. 2021 [31], Norway, 1995–2016	Case-control	BreastScreen Norway	Multi	Mean 5.2	DCIS: 131 DCIS + IBC: 80	211	Cases: 59.8 (55.2–65.7) controls: 59.4 (54.1–63.7)	Cases: 2 (1.3–3.8) Controls: 2 (0.9–3.5)	N/A	N/A
Nishimura et al. 2004 [33], Japan, 1995–2001	Cohort	Cancer institute	Single	N/A	DCIS: 124 IBC: 33	NS	NS (NS)	NS	N/A	N/A
Rauch et al. 2016 [34], US, 1996–2009	Cohort	University hospital	Single	Mean 7 (range 1–16)	1438^a^	NS	55 (11)	2.25 (2.5)	N/A	N/A
Rominger et al. 2015 [35], US, 2002–2003	Cohort	Academic tertiary-care institution	Single	Mean 6.8	DCIS: 57 IBC: 42	NS	NS (NS)	NS	IHC NOS	NS
Stomper et al. 2003 [37], US, 1991–2001	Cohort	Mammography center and a multidisciplinary breast clinic in a cancer institute	Multi	N/A	DCIS: 198 DCIS + IBC: 96 IBC: 10	304	Median: 54 (29–86)	NS	N/A	N/A
Szynglarewicz et al. 2016 [38], Poland, 2009–2014	Cohort	University hospital	Single	N/A	127	127	59.6 (50–69)	NS (NS)	N/A	N/A
Tabar et al. 2011 [40], Sweden, 1977–2007	Cohort	University hospitals	Multi	Falun: mean 5, Roanoke: mean 3	DCIS: 498 IBC: 353	NS	NS (NS)	NS (0.1–1.4)	N/A	N/A
Tan et al. 2000 [41], Singapore, 1993–1996	Cohort	Singapore breast screening project	Multi	N/A	32	25	Median: 57 (51–66)	NS	N/A	N/A
Thurfjell et al. 2002 [43], Sweden, 1988–1994	Cohort	University hospital	Single	N/A	DCIS: 57 IBC: 71	DCIS: 50 IBC: 29	Median: 61 (33–87)	NS	N/A	N/A
Wang et al. 2019 [44], China, 2012–2015	Cohort	University hospital	Single	N/A	60	60	DCIS: 48 (11) DCISM: 46 (8)	DCIS 2.1 (1.5) DCISM 3.4 (1.5)	N/A	N/A
Woodard et al. 2019 [45], US, 2012–2017	Cohort	University hospital	Single	N/A	58	58	54.8 (9.7)	NS (NS)	N/A	N/A
Zhang et al. 2021 [46], China, 2016–2020	Cohort	University hospital	Single	N/A	395	171	Median: 50 (27–87)	DCIS: 67.8% < 2.7 DCIS-MI: 42.1% < 2.7	N/A	N/A
Zhou et al. 2017 [47], Sweden, 1986–2004	Cohort	From Uppland and Västmanland counties	Multi	Mean 15.4	310	NS	57.5 (NS)^a^	NS (NS)	Tma data, IHC dako HercepTest; SISH NOS	ER/PR: > 10% HER2: SISH+ or IHC 3+
Zhou et al. 2014 [48], Sweden, 2005–2006	Cohort	Academic hospital and central hospital	Multi	N/A	DCIS: 32 DCIS + IBC: 42	NS	Majority (60.8%) > 55 years^a^	NS	N/A	N/A

**Study characteristics**		**Assessment clinicopathological factors**			**Assessment calcification morphology**				
		**(molecular subtype)**		**Other**					
*Ref., country, recruitment period*	*(Observational) study design*	*Assay grade*	*Number of readers*	*Assay*	*Imaging system (manufacturer)*	*Method of detection*	*No. of readers*	*Blinded*	*Calcification classification system*
Avdan Aslan et al. 2021 [16], Turkey, 2007–2013	Cohort	Modified Bloom-Richardson Grading System	1 (reviewed)	Comedonecrosis	Selenia (Hologic)	NS	2 (reviewed)	Yes	BIRADS 5th edition (2013)
Bae et al. 2013 [17], South Korea, 2006–2011	Cohort	N/A	N/A	N/A	Senographe 2000D (GE Healthcare); Selenia (Hologic)	NS	3 (reviewed)	Yes	BIRADS 4th edition (2003)
Bagnall et al. 2001 [18], England, 1994–1999	Cohort	N/A	N/A	Invasion	NS	SD and non-SD	NS	NS	NS (punctate, granular or linear)
Barreau et al. 2005 [19], France, 1980–1999	Cohort	WHO breast tumor classification 6th edition	NS	Comedocarcinoma, necrosis	NS	NS	1 (reviewed)	NS	BIRADS (1993–2003) 1st - 4th edition
de Roos et al. 2006 [21], The Netherlands, 1991–2003	Cohort	N/A	Reclassified by 1	Margin status	NS	SD and non-SD	1 (reclassified)	Yes	Holland R, Hendriks JHCL (1994)
de Roos et al. 2004 [20], the Netherlands, 1986–2000	Cohort	EPWG and Van Nuys	1 (reviewed)	N/A	NS	SD and non-SD	1 (reviewed)	Partial, to histopathological subtype	Holland R, Hendriks JHCL (1994)
Dinkel et al. 2000 [22], Germany, 1993–1998	Cohort	Nuclear malignancy grade and EORTC grading	1 (reviewed)	N/A	Mammomat 2 (Siemens)	NS	≥ 1 (reviewed)	Yes	Holland R, Hendriks JHCL (1994)
Evans et al. 2010 [23], UK, NS	Cohort	Cytonuclear grade	NS (pathology dataset)	Microscopic tumor size	NS	SD	NS	No	NS (Groups: casting/linear, granular/irregular, or punctate)
Hofvind et al. 2011 [24], Norway, 1995–2007	Cohort	Van Nuys	1 (reviewed)	Distribution of calcifications	Rotolux/Planilux 400 (Siemens); Advanced Workstation 4.4 (GE Healthcare)	SD	4 (reviewed)	yes, to side, site, size, clinical findings, and further histologies Information	Modified BIRADS 4th edition (2003)
Holmberg et al. 2013 [25], Sweden, 1987–1999	Case-cohort study within a randomized trial	Nuclear grade	NS	Local recurrence in the ipsilateral breast, age, necrosis, tumor size	NS	SD and non-SD	1 (reviewed)	Yes	Tabar, L (2005)
Kessar et al. 2002 [26], England, 1989–1996	Cohort	Van Nuys	NS	N/A	NS	SD and non-SD	3 (reviewed)	NS	NS (groups: pleomorphic, linear branching, punctate)
Kim et al. 2015 [27], South Korea, 2007–2013	Cohort	N/A	N/A	N/A	Selenia (Hologic)	SD	2 (reviewed)	Yes	BIRADS 5th edition (2013)
Kong et al. 2020 [28], China, January 2018–December 2018	Cohort	NS	NS	N/A	Selenia (Hologic)	SD and non-SD	NS	NS	BIRADS 5th edition (2013)
Lee et al. 2000 [29], US, 1992–1999	Cohort	N/A	N/A	Invasion, defined as clear evidence of carcinoma in the stroma	NS	SD	1 (reviewed)	Yes	BIRADS 1st edition (1993)
Lee et al. 2021 [30], South Korea, 2009–2019	Cohort	Van Nuys	NS	Comedonecrosis, ki67	NS	NS	2	Yes	BIRADS 5th edition (2013)
Lilleborge et al. 2021 [31], Norway, 1995–2016	Case-control	N/A	N/A	Invasion, defined as breast cancer > 6 months after the diagnosis of DCIS	NS	SD	1 (reviewed)	Yes	BIRADS 5th edition (2013)
Nishimura et al. 2004 [33], Japan, 1995–2001	Cohort	N/A	N/A	Invasion	NS	SD	NS	NS	BIRADS 3rd edition (1998)
Rauch et al. 2016 [34], US, 1996–2009	Cohort	NS	1 of 11 (reviewed)	Presence of comedonecrosis, multicentricity and local recurrence	NS	NS	2 (1 original reading and 1 rereading)	NS	BIRADS 5th edition (2013)
Rominger et al. 2015 [35], US, 2002–2003	Cohort	Van Nuys	NS	Local recurrence	NS	SD	NS	NS	BIRADS 5th edition (2013)
Stomper et al. 2003 [37], US, 1991–2001	Cohort	N/A	N/A	Invasion, extent of invasion and calcification size	High-resolution film-screen mammography including orthogonal magnification projections	SD	NS	Yes	Holland R, Hendriks JHCL (1994)
Szynglarewicz et al. 2016 [38], Poland, 2009–2014	Cohort	Nuclear grade based on College of American Pathologists	NS	Comedonecrosis, age (only *p* value), calcification distribution	NS	SD	3 (1 reviewed)	Yes	Tabar, L (2005)
Tabar et al. 2011 [40], Sweden, 1977–2007	Cohort	N/A	N/A	Invasion	NS	SD and non-SD	1	Yes	Tabar, L (2004)
Tan et al. 2000 [41], Singapore, 1993–1996	Cohort	Nuclear pleomorphism based on Lagios	NS	Necrosis	NS	SD	NS	NS	Tabar, L (1997)
Thurfjell et al. 2002 [43], Sweden, 1988–1994	Cohort	Nuclear grade based on Elston and Ellis	1	Invasion	NS	SD and non-SD	1 (reviewed)	NS	Tabar, L (1983) and BIRADS 3rd edition (1998)
Wang et al. 2019 [44], China, 2012–2015	Cohort	N/A	N/A	Micro invasion, defined as a microscopic focus of invasion ≤ 1 mm in the longest diameter within an area of DCIS	Planmed Nuance (Planmed)	NS	2 (reviewed)	Yes	BIRADS 5th edition (2013)
Woodard et al. 2019 [45], US, 2012–2017	Cohort	N/A	N/A	Local recurrence (the Oncotype DX Breast DCIS assay)	NS	NS	1 (reviewed)	Yes	BIRADS 5th edition (2013)
Zhang et al. 2021 [46], China, 2016–2020	Cohort	N/A	N/A	Microinvasion defined as the extension of cancer cells beyond the basement membrane into the adjacent tissues with no focus > 0.1 cm in the greatest dimension	Selenia (Hologic)	SD and non-SD	2	NS	BIRADS 5th edition (2013)
Zhou et al. 2017 [47], Sweden, 1986–2004	Cohort	EORTC	NS	Age, neoductgenesis, Tenascin-C, proliferation and recurrence	NS	SD and non-SD	2 (reviewed)	Yes	Tabar, L (2004)
Zhou et al. 2014 [48], Sweden, 2005–2006	Cohort	N/A	N/A	Neoductgenesis	NS	NS	2	Yes	Tabar, L (2004)

*Abbreviations*: *DCIS* Ductal carcinoma in situ, *IBC* Invasive breast cancer, *N/A* Not available (e.g., if not related to CMD), *NS* Not specified, *NOS* Not otherwise specified, *SD* Screen detected

^a^Calculated for subgroups

### Reported clinicopathological factors in relation to CMDs

A total of 29 studies investigated 17 distinct factors concerning CMDs (Table 2), with 28 studies assessing non-prognostic outcomes, including high grade (*n* = 16), (micro)invasion (*n* = 8), (comedo)necrosis (*n* = 7), HER2 overexpression (*n* = 6), ER positivity (*n* = 6), age (*n* = 3), Ki67 or proliferation (*n* = 2), histological size (*n* = 2), neoductgenesis (*n* = 2), calcification distribution (*n* = 2), margin status (*n* = 1), comedocarcinoma (*n* = 1), multicentricity (*n* = 1), tenascin-C (*n* = 1), and Oncotype DX score (*n* = 1). Furthermore, five studies assessed prognostic outcomes including recurrence (*n* = 4) and DCIS progression to IBC (*n* = 1).

**Table 2:** Overview of clinicopathological factors that were assessed in the studies

**Clinicopathological factors**	**First author, year of publication (reference)**	**No. of studies**		**No. included in meta-analysis**
		**Assessed factors**	**Statistically significant finding**	
Grade	Avdan Aslan, 2021 [16]; Barreau, 2005 [19]; de Roos, 2006 [20]; Dinkel, 2000 [22]; Evans, 2010 [23]; Hofvind, 2011 [24]; Holmberg, 2013 [25]; Kessar, 2002 [26]; Kong, 2020 [28]; Lee, 2021 [30]; Rauch, 2016 [34]; Rominger, 2015 [35]; Szynglarewicz, 2016 [38]; Tan, 2000 [41]; Thurfjell, 2002 [43]; Zhou, 2017 [47]	16	8	11 studies in meta-analysis
Presence (micro) invasive breast cancer	Bagnall, 2001 [18]; Lee, 2000 [29]; Nishimura, 2004 [33]; Stomper, 2003 [37]; Tabar, 2011 [40]; Thurfjell, 2002 [43]; Wang, 2019 [44]; Zhang, 2021 [46]	8	1	5 studies in meta-analysis
(Comedo)necrosis	Avdan Aslan, 2021 [16]; Barreau, 2005 [19]; Holmberg, 2013 [25]; Lee, 2021 [30]; Rauch, 2016 [34]; Szynglarewicz, 2016 [38]; Tan, 2000 [41]	7	4	5 studies in meta-analysis
Her2 overexpression	Avdan Aslan, 2021 [16]; Bae, 2013 [17]; Kim, 2015 [27]; Lee, 2021 [30]; Rominger, 2015 [35]; Zhou, 2017 [47]	6	3	4 studies in meta-analysis
ER positivity	Avdan Aslan, 2021 [16]; Bae, 2013 [17]; Kim, 2015 [27]; Lee, 2021 [30]; Rominger, 2015 [35]; Zhou, 2017 [47]	6	3	4 studies in meta-analysis
Recurrence^a^	Holmberg, 2013 [25]; Rauch, 2016 [34]; Rominger, 2015 [35]; Zhou, 2017 [47]	4	1	Groups not comparable for meta-analysis
Age	Holmberg, 2013 [25]; Szynglarewicz, 2016 [38]; Zhou, 2017 [47]	3	0	Groups not comparable for meta-analysis
Ki67/proliferation	Lee, 2021 [30]; Zhou, 2017 [47]	2	0	Groups not comparable for meta-analysis
Histological size	Evans, 2010 [23]; Holmberg, 2013 [25]	2	1	Groups not comparable for meta-analysis
Neoductgenesis	Zhou, 2017 [47]; Zhou, 2014 [48]	2	1	Groups not comparable for meta-analysis
Calcification distribution	Hofvind, 2011 [24]; Szynglarewicz, 2016 [38]	2	1	Groups not comparable for meta-analysis
Margin status	de Roos, 2004 [21]	1	1	Meta-analysis N/A
Comedocarcinoma	Barreau, 2005 [19]	1	1	Meta-analysis N/A
Multicentricity	Rauch, 2016 [34]	1	0	Meta-analysis N/A
Tenascin-C	Zhou, 2017 [47]	1	1	Meta-analysis N/A
Oncotype DX score	Woodard, 2019 [45]	1	1	Meta-analysis N/A
Progression to invasive disease^a^	Lilleborge, 2021 [31]	1	1	Meta-analysis N/A

^a^Prognostic outcomes

### Data synthesis and meta-analysis

**Fig. 2:**
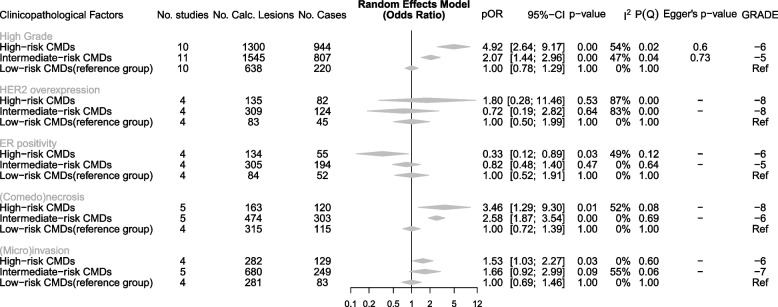
The meta-analysis results for each clinicopathological factor in a forest plot. For the calcification morphology descriptor (CMD) risk groups, the pooled odds ratios (pORs), 95% confidence intervals and associated *p*-values are shown. Furthermore, associated heterogeneity measures (*I*^2^, *P*(*Q*)) and publication bias (Egger’s *p*-value), as well as certainty of evidence summarized in the GRADE score are given. The low-risk CMDs served as a reference. Per CMD risk-group, details on the studies (number, number of calcified lesions, and number of cases) are given

Out of the 17 clinicopathological factors reported across 29 studies, 14 factors were significantly association in at least one study (Table 2). A meta-analysis was conducted for five clinicopathological factors, deemed sufficiently homogeneous across 20 studies (Fig. 2): high grade (*n* = 11), HER2 overexpression (*n* = 4), ER positivity (*n* = 4), (comedo)necrosis (*n* = 5), and the presence of (micro)invasion) (*n* = 5). The meta-analysis shows the aggregated results for low-, intermediate-, and high-risk CMDs concerning the clinicopathological factors.

High-risk CMDs demonstrated a significant association with four clinicopathological factors including high grade (pOR, 4.92; 95% CI, 2.64–9.17), (comedo)necrosis (pOR, 3.46; 95% CI, 1.29–9.30), (micro)invasion (pOR, 1.53; 95% CI, 1.03–2.27), and ER positivity (pOR, 0.33; 95% CI, 0.12–0.89). High-risk CMDs were negatively associated with ER positivity, indicating a reduced incidence of ER positivity in high-risk versus low-risk CMDs.

Intermediate-risk CMDs were significantly associated with high grade (pOR, 2.07; 95% CI, 1.44–2.96) and (comedo)necrosis (pOR, 2.58; 95% CI, 1.87–3.54), while showing an increased pOR of 1.66 (95% CI, 0.92–2.99) with a *p*-value of 0.09 for (micro)invasion.

Heterogeneity measures *I*^2^ and *P*(*Q*) revealed inconsistencies in the estimates reported in the included studies concerning high grade (*I*^2^, *P*(*Q*): 54%, *p* = 0.002 for high-risk and 47%, *p* = 0.04 for intermediate-risk CMDs), ER positivity (*I*^2^, *P*(*Q*): 49%, *p* = 0.12 for high-risk CMDs), comedo(necrosis) (*I*^2^, *P*(*Q*): 52%, *p* = 0.08 for high-risk CMDs), and invasion (*I*^2^, *P*(*Q*): 55%, *p* = 0.06 for intermediate-risk CMDs).

Neither high-risk CMDs (pOR, 1.80; 95% CI, 0.28–11.46) nor intermediate-risk CMDs (pOR, 0.72; 95% CI, 0.19–2.82) were significantly associated with HER2. One contributing study by Zhou et al. [47] reported odds ratios below one, indicating a reduced risk. A considerable discrepancy existed between odds ratios calculated from the different included studies, reflected in the heterogeneity measures *I*^2^ with > 83% and *p*(*Q*) <  = 0.001.

Egger’s test was not significant, indicating that there was no publication bias for high grade, while publication bias was not determined due to the small sample sizes for the other outcomes.

### Certainty of evidence

According to the GRADE tool approach, the certainty of evidence for all outcomes can be rated as low (Supplementary Table S3), as the studies assessed associations through observations. The calculated GRADE score denoted the level of insufficient evidence or bias across five domains (risk of bias according to the QUIPS tool, heterogeneity, indirectness, imprecision and publication bias). The highest GRADE score of −11 for (high-risk and intermediate-risk CMDs combined) was identified for ER positivity and high grade, indicating the highest level of evidence. The GRADE score for the other outcomes were as follows: invasion (−13), (comedo)necrosis (−14), and HER2 overexpression (−16). The next section evaluates the risk of bias across the selected studies in detail using the QUIPS tool.

### Risk of bias per QUIPS domain

**Fig. 3:**
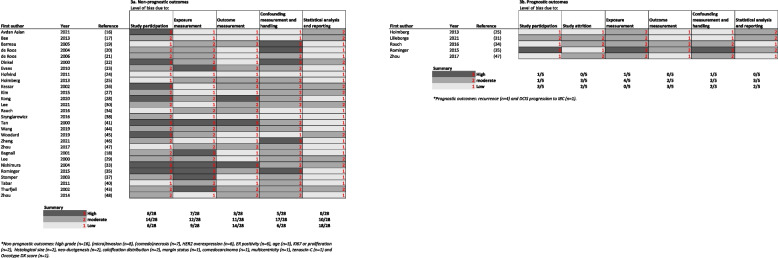
Risk of bias per QUIPS domain for each individual study with (**a**) non-prognostic outcome(s) and (**b**) prognostic outcome(s)

To further understand the reliability of the included studies, a thorough assessment of bias was conducted using the QUIPS tool. The risk of bias was assessed across five study domains, namely study participation, exposure measurement, outcome measurement, study confounding, and statistical analysis and reporting. For studies measuring prognostic outcomes a sixth domain, study attrition, was also evaluated (Fig. 3).

Across the 29 studies, five out of 170 individual rated QUIPS domains exhibited a low-high discrepancy between the two reviewers in their rating of bias. Following consultation with the third reviewer (A.W.B.D.), these domains were assigned a moderate risk of bias rating. This suggests that the discrepancies in the assessment of the bias using the QUIPS tool were limited.

The study participation domain revealed eight studies with a high risk of bias and 15 with a moderate risk in either prognostic or non-prognostic outcomes. The downgrading of studies was primarily attributed to small sample sizes and inadequate description of study groups, data collection criteria, and methods or reasons for missing data.

In the exposure domain, seven studies exhibited a high risk of bias, while 13 demonstrated a moderate risk. The downgrading mainly resulted from situations where only one reader determined the CMDs, or when crucial details were omitted, such as whether the readers were blinded to the outcome and how consensus was achieved between readers.

The outcome measurement domain indicated three studies with a high risk of bias and 11 studies with a moderate risk. High-risk studies were characterized by a severe lack of detail regarding the definition and method of measuring the outcome variable. Moderate-risk studies contained insufficient information on the measurement of outcome variables and, if applicable, blinding of reviewers.

With regard to the confounding domain, most individual studies did not adjust their results for potential confounders. Five studies were rated as having a high risk of bias in this domain and 18 as having a moderate risk of bias. High-risk studies failed to account for potential confounding through matching, stratification, or the initial assembly of comparable groups. Prognostic studies were rated as moderate or high risk when they did not adjust for treatment or age in their statistical analyses. Studies with a design that somewhat limited the risk of confounding were rated as moderate risk.

The statistical analysis and reporting domain predominantly displayed a low risk of bias. However, in thirteen studies, this domain was rated as moderate, because the analysis was not powerful enough to prove or disprove the hypothesis. This occasionally occurred for individual CMD groups, e.g., when chi-square tests were applied to small sample sizes.

In the study attrition domain, three prognostic studies were rated as having a moderate risk of bias because the follow-up or characteristics of women who completed the study and those who did not were not described.

Notably, the average risk of bias was significantly higher in the exposure measurement (*p* = 0.01) and confounding (*p* = 0.025) domains for studies published before 2010 compared to those published after 2010.

## Discussion

### Data synthesis and meta-analysis

This systematic review comprehensively synthesizes the existent literature examining the associations between calcification morphological descriptors (CMDs) and clinicopathological factors in women diagnosed with DCIS with the aim of distinguishing high-risk from low-risk DCIS lesions based on CMDs, which may aid clinical decision making. A total of twenty-nine studies were identified, evaluating 17 clinicopathological factors, of which five were deemed appropriate for meta-analysis.

The meta-analysis revealed a significant association between fine linear calcifications, i.e., the high-risk group and features of aggressiveness including high grade, presence of (comedo)necrosis, and (micro)invasion. An inverse association was observed with ER positivity.

Intermediate-risk CMDs, i.e., coarse heterogeneous and fine pleomorphic calcifications, were significantly associated with high-grade and (comedo)necrosis. The associations were generally similar to those of the fine linear calcifications, albeit to a lesser extent. The presence of calcifications in DCIS is thought to be due to active secretion of calcium into the ducts by (malignant) epithelial cells in non-comedocarcinoma and calcification of necrotic debris in comedocarcinoma [58]. The observed association between fine linear calcifications and the presence of (comedo)necrosis, high grade and (micro)invasion might be attributed to the rapid growth and common cell death that occurs within poorly differentiated DCIS, culminating in calcification deposition along the ductal structures and their linear appearance on mammography. Fine linear calcifications may thus be more associated to more aggressive malignancy, as their linear appearance suggests a duct lumen filled with calcified necrotic debris [58].

With respect to HER2 overexpression, a positive association with high-risk CMDs was found, whereas a negative association was identified with intermediate-risk CMDs; however, neither association was statistically significant. The results were inconsistent, due to one study [47] presenting contradictory findings compared to other studies [16, 17, 27] that reported on HER2. The study’s characteristics did not provide a clear explanation for this discrepancy, with the only apparent difference being the use of tissue microarrays rather than tissue resections. Nevertheless, a meta-analysis conducted in 2013 by Elias et al. [59] which aimed to identify imaging features of HER2 overexpression in multiple imaging modalities and included IBC lesions next to DCIS lesions, discovered a significant association between HER2 overexpression and high-risk CMDs on mammography. For intermediate-risk calcifications, they found a positive, non-significant association with pleomorphic calcifications and no association with coarse calcifications.

Considering the prior findings that ER-positive and HER2-negative (luminal) breast cancers are generally less aggressive than ER-negative and HER2-positive invasive breast cancers [60], the associations between linear calcifications with HER2 overexpression and negative association with ER-positivity in our review were not unexpected. Further research is warranted to elucidate the role of receptor subtypes in the risk profile of calcifications and associated lesions.

Twelve other clinicopathological factors were examined in relation to CMD, in addition to the ones that were suitable for inclusion in the meta-analysis, and several studies reported either significant or non-significant associations for these factors. Among these factors, the one related to the risk of recurrence including the Oncotype DX score showed significant association in two out of five studies. However, additional cohorts and standardized methods are required to validate the evidence on these factors.

Our comprehensive analysis suggests a potential association between CMDs and the aggressiveness of lesions, particularly in the progression from DCIS to IBC. This conclusion is supported by O’Grady et al. [61], and Tot et al. [62], who presented evidence from a selection of important clinicopathological factors and outcomes in their respective non-systematic reviews. However, it is essential to note that these are narrative reviews without described strategies to identify and mitigate reporting bias. The majority of the referenced studies were centered on IBC lesions, with some of them solely examining presence of calcifications or comparing specific calcification morphologies to their absence. Moreover, Tot et al. [62] grouped different calcifications into two main categories: those mostly occupying the ducts (including casting-type and skipping stone-like calcifications) and those predominantly involving the TDLUs (including crushed stone-like and powdery calcification). This resulted in less detailed information about different mammographic CMDs. To confirm the significance of CMDs as a prognostic biomarker, CMDs should be studied in terms of prognostic outcomes, such as survival or recurrence rates, next to clinicopathological factors.

### Certainty of evidence and sources of bias

Uncertainty in the findings of the meta-analysis were revealed and the most common sources of bias in the relevant articles using the GRADE approach and the QUIPS tool. The primary source of bias originated from the study participation domain due to low sample size and inadequately described study groups. The majority of the included studies utilized data from hospital or national registries, which could have influenced the found estimates. Retrospective registry-based studies depend on the quality, size, and completeness of relevant variables and features of the used registries. Frequently, these variables were not mentioned or properly described.

The exposure domain was also frequently rated as moderate or high risk of bias because CMD determination was often conducted by a single reader, given that the assessment of these qualitative descriptors is prone to inter- and intra-observer variability. While radiologists strongly agree on the presence of calcifications, they agree to a lesser degree on the classification of the observed calcification morphology [63]. Further standardization of the descriptors is therefore essential for using CMDs in medical decision-making. Specifically, methods that can extract high-quality features from radiological images, such as radiomics and deep learning, hold the potential to accurately discriminate between calcifications associated with low- and high-risk DCIS [64–66]. In addition to AI and radiomics, other imaging modalities and image enhancement techniques (e.g., noise reduction and contrast manipulation) could be considered depending on accessibility, numbers, and costs, as mammography images suffer from low contrast and background, making breast cancer diagnosis challenging. Using accurate prediction models could facilitate the assessment of different calcification types more reliably in a more objective manner, overcoming the substantial inter-reader variability among radiologists. Ultimately, this may aid in the clinical management of lesions associated with such calcifications.

Bias due to inter-reader variability could also have occurred in the outcome domain for the clinicopathological factor grade, meaning that both radiomics and pathomics methods might improve risk stratification of calcifications.

Concerning the confounding domain, most studies reported on the morphology of calcifications in isolation, not considering other descriptors that can aid in further risk stratification and control for confounding factors such as distribution, size, and clinicopathological factors. Some studies also assessed distribution, as this is another calcification descriptor often used in clinical practice, but not in combination with morphology. Hence, the results from this review reflect univariable associations only, which can lead to biased estimates and incorrect conclusions if relevant covariates are omitted.

The studies published after 2010 had significantly lower risk of bias scores in the exposure and confounding domains as compared to the older studies from before 2010, indicating an improvement in study and evaluation methods for this research question.

## Limitations and strengths

As with the majority of systematic reviews, the design of the current study is subject to potential limitations [67]. Systematic reviews employ a retrospective, observational research design, and as such are susceptible to systematic and random error. The majority of known errors in systematic reviews arise during the selection and reporting stages [68]. To mitigate the risk of these errors, an information specialist (S.M.) was consulted in advance to define all the steps and judgments in the systematic review process and to conduct the search of the articles. Furthermore, M.M.L. and S.D. piloted the screening, quality assessment, and data extraction process to improve accurate interpretation and discussed discrepancies between their results with A.W.B.D. or the whole team.

Studies with a high risk of bias according to the QUIPS tool were not excluded in our meta-analysis given that the risk of bias was relatively high for all studies, predominantly due to small sample sizes and inconsistencies in the evaluation and registration of calcifications. These biases have affected the reported pOR estimates, as well as the performance of tests of heterogeneity, publication bias, and other sample size effects summarized in the GRADE score for each clinicopathological factor and calcification group. For a more nuanced interpretation of the meta-analysis results, the ORs and QUIPS scores per domain for each study were reported.

Nonetheless, this is the first systematic review and meta-analysis to assess the association between mammographic CMDs and clinicopathologic factors in women with DCIS. The primary strength of such a meta-analysis lies in its capacity to enhance the identification of associations and uncover the sources of heterogeneity between reported estimates across studies. Indeed, using the QUIPS tool, we identified the most frequently occurring biases in included studies by assessing the association between CMDs and clinicopathological factors in a standardized manner.

In conclusion, our meta-analysis demonstrates that specific mammographic calcification morphologies are related to clinicopathological factors associated with lesion aggressiveness in women with DCIS.

This systematic review also showed a high risk of bias and heterogeneity between studies. Therefore, these findings need to be verified through high-quality studies that use homogeneous cohorts and standardized, reliable calcification assessment systems. Future radiomics and deep learning studies may help in the extraction of relevant calcification features that can extract prognostic information in DCIS lesions and, ultimately, in making the distinction between high-risk and low-risk DCIS lesions and reducing overtreatment of DCIS.

### Supplementary Information

**Additional file 1: Supplementary Table S1.** S1a Table: Search strategy used in Medline via Ovid. S1b Table: Search strategy used in Embase. S1c Table: Search strategy used in Web of Science.

**Additional file 2: Supplementary Table S2.** QUIPS master form.

**Additional file 3: Supplementary Table S3.** Grade assessment.

**Additional file 4: Supplementary Table S4.** Alignment of BIRADS and Non-BIRADS calcification morphology descriptors.

**Additional file 5: Supplementary Table S5.** Excluded articles after full text screening.

## Data Availability

The authors confirm that the data supporting the findings of this study are available within the article and its supplementary material. Raw data that supports the findings of this study are available from the corresponding author, upon reasonable request.

## Abbreviations

BI-RADS: Breast Imaging-Reporting and Data System
CMD: Calcification morphology descriptor
DCIS: Ductal carcinoma in situ
GRADE: Grading of Recommendations, Assessment, Development, and Evaluations
IBC: Invasive breast cancer
pOR: Pooled odds ratio
QUIPS: Quality in Prognostic Studies
