# Supplementary material for: Clinicopathological and prognostic value of calcification morphology descriptors in ductal carcinoma in situ of the breast: a systematic review and meta-analysis
Source: Insights Imaging. 2023 Dec 5;14:213. doi: 10.1186/s13244-023-01529-z (PMC10697924; doi:10.1186/s13244-023-01529-z)
Supplement: Supplementary file 1 — Additional file 1: Supplementary Table S1. S1a Table: Search strategy used in Medline via Ovid. S1b Table: Search strategy used in Embase. S1c Table: Search strategy used in Web of Science. [file 13244_2023_1529_MOESM1_ESM.docx]

**Clinicopathological and prognostic value of calcification morphology descriptors in Ductal Carcinoma in Situ of the breast: a systematic review and meta-analysis**

**ELECTRONIC SUPPLEMENTARY MATERIAL**

**S1a Table: Search strategy used in Medline via Ovid**

| # | Query |
| --- | --- |
| 1 | calcinosis/ or "Calcification, Physiologic"/ |
| 2 | (calcinos* or microcalcinos* or calcification* or microcalcif* or "calcinotic deposit*" or "calcium deposit*").ti,ab,kf. |
| 3 | or/1-2 [calcinosis] |
| 4 | exp mammography/ |
| 5 | calcinosis/dg |
| 6 | (mammogra* or mastograph*).ti,ab,kf. |
| 7 | (breast* adj3 (screening or imag*)).ti,ab,kf. |
| 8 | or/4-7 [mammography] |
| 9 | ("bi-rads" or birads).ti,ab,kf. |
| 10 | ("breast imag*" adj1 reporting adj2 "data system*").ti,ab,kf. |
| 11 | or/9-10 [BIRADS] |
| 12 | 3 and (8 or 11) [calcinosis AND (mammography OR BIRADS)] |
| 13 | "Carcinoma, Intraductal, Noninfiltrating"/ |
| 14 | (DCIS or CDIS or "ductal carcinoma in situ" or "stage zero breast cancer*" or "intra-ductal carcinoma*" or "intraductal carcinoma*" or "ductal carcinoma*").ti,ab,kf. |
| 15 | (("pre-invasive" or "preinvasive" or "non-invasive" or "noninvasive" or "non-infiltrating" or "noninfiltrating") adj1 ("ductal carcinoma*" or "breast carcinoma*" or "breast tumor*" or "breast tumour*" or "breast cancer*")).ti,ab,kf. |
| 16 | (("mammary" or "ductal") adj1 ("intra-epithelial" or intraepithelial) adj1 neoplas*).ti,ab,kf. |
| 17 | or/13-16 [DCIS] |
| 18 | exp "breast neoplasms"/ |
| 19 | ((breast* or mamma*) adj3 (cancer* or neoplasm* or carcinoma* or adenocarcinoma* or malignan* or tumor* or tumour* or metasta*)).ti,ab,kf. |
| 20 | or/18-19 [(invasive) breast cancer] |
| 21 | 17 or 20 [DCIS OR breast cancer] |
| 22 | 12 and 21 [(calcinosis AND (mammography or BIRADS)) AND (DCIS OR breast cancer)] |
| 23 | limit 22 to yr="2000-Current" |

**** Asterisk used to capture multiple word ending (e.g. calcifying, calcification)***

**S1b Table: Search strategy used in Embase**

| # | Query |
| --- | --- |
| 1 | (calcification/de OR calcinosis/exp OR microcalcification/de OR "soft tissue calcification"/de) |
| 2 | (calcinos* OR microcalcinos* OR calcification* OR microcalcif* OR "calcinotic deposit*" OR "calcium deposit*"):ti,ab,kw |
| 3 | #1 OR #2 |
| 4 | 'mammography'/exp |
| 5 | (mammogra* OR mastograph*):ti,ab,kw |
| 6 | (breast* NEAR/3 (screening OR imag*)):ti,ab,kw |
| 7 | #4 OR #5 OR #6 |
| 8 | 'breast imaging reporting and data system'/exp |
| 9 | ("bi-rads" OR birads):ti,ab,kw |
| 10 | ('breast imag*' NEAR/1 reporting NEAR/2 'data system*'):ti,ab,kw |
| 11 | #8 OR #9 OR #10 |
| 12 | #3 AND (#7 OR #11) |
| 13 | 'intraductal carcinoma'/exp |
| 14 | ((DCIS OR CDIS OR "ductal carcinoma in situ" OR "stage zero breast cancer*" OR "intra-ductal carcinoma*" OR "intraductal carcinoma*" OR "ductal carcinoma*"):ti,ab,kw) |
| 15 | (("pre-invasive" OR "preinvasive" OR "non-invasive" OR "noninvasive" OR "non-infiltrating" OR "noninfiltrating") NEAR/1 ("ductal carcinoma*" OR "breast carcinoma*" OR "breast tumor*" OR "breast tumour*" OR "breast cancer*")):ti,ab,kw |
| 16 | (("mammary" OR "ductal") NEAR/1 ("intra-epithelial" OR intraepithelial) NEAR/1 (neoplas*)):ti,ab,kw |
| 17 | #13 OR #14 OR #15 OR #16 |
| 18 | 'breast cancer'/exp |
| 19 | ((breast* OR mamma*) NEAR/3 (cancer* OR neoplasm* OR carcinoma* OR adenocarcinoma* OR malignan* OR tumor* OR tumour* OR metasta*)):ti,ab,kw |
| 20 | #18 OR #19 |
| 21 | #17 OR #20 |
| 22 | #12 AND #21 |
| 23 | #22 AND [2000-2023]/py |
| 24 | #23 NOT ('conference abstract'/it OR 'conference paper'/it OR 'conference review'/it) |

**** Asterisk used to capture multiple word ending (e.g. calcifying, calcification)***

**S1c Table: Search strategy used in Web of Science**

| # | Query |
| --- | --- |
| 1 | TS=(calcinos* OR microcalcinos* OR calcification* OR microcalcif* OR "calcinotic deposit*" OR "calcium deposit*") |
| 2 | TS=(mammogra* OR mastograph*) |
| 3 | TS=(breast* NEAR/3 (screening OR imag*)) |
| 4 | #2 OR #3 |
| 5 | TS=("bi-rads" OR birads) |
| 6 | TS=("breast imag*" NEAR/1 reporting NEAR/2 "data system*") |
| 7 | #5 OR #6 |
| 8 | #1 AND (#4 OR #7) |
| 9 | TS=(DCIS OR CDIS OR "ductal carcinoma in situ" OR "stage zero breast cancer*" OR "intra-ductal carcinoma*" OR "intraductal carcinoma*" OR "ductal carcinoma*") |
| 10 | TS=(("pre-invasive" OR "preinvasive" OR "non-invasive" OR "noninvasive" OR "non-infiltrating" OR "noninfiltrating") NEAR/1 ("ductal carcinoma*" OR "breast carcinoma*" OR "breast tumor*" OR "breast tumour*" OR "breast cancer*")) |
| 11 | TS=(("mammary" OR "ductal") NEAR/1 ("intra-epithelial" OR intraepithelial) NEAR/1 (neoplas*)) |
| 12 | #9 OR #10 OR #11 |
| 13 | TS=((breast* OR mamma*) NEAR/3 (cancer* OR neoplasm* OR carcinoma* OR adenocarcinoma* OR malignan* OR tumor* OR tumour* OR metasta*)) |
| 14 | #12 OR #13 |
| 15 | #8 AND #14 |
| 16 | #15 AND PY=(2000-2023) |
| 17 | #16 NOT DT=(Meeting Abstract OR Meeting Summary OR Proceedings Paper) |

**** Asterisk used to capture multiple word ending (e.g. calcifying, calcification)***
