# Supplementary material for: Clinicopathological and prognostic value of calcification morphology descriptors in ductal carcinoma in situ of the breast: a systematic review and meta-analysis
Source: Insights Imaging. 2023 Dec 5;14:213. doi: 10.1186/s13244-023-01529-z (PMC10697924; doi:10.1186/s13244-023-01529-z)
Supplement: Supplementary file 4 — Additional file 4: Supplementary Table S4. Alignment of BIRADS and Non-BIRADS calcification morphology descriptors. [file 13244_2023_1529_MOESM4_ESM.docx]

**Clinicopathological and prognostic value of calcification morphology descriptors in Ductal Carcinoma in Situ of the breast: a systematic review and meta-analysis**

**ELECTRONIC SUPPLEMENTARY MATERIAL**

| Supplementary table 4 *Alignment of* *BIRADS and Non-BIRADS calcification morphology descriptors* | | | | | | |
| --- | --- | --- | --- | --- | --- | --- |
|  |  |  |  |  |  |  |
| Reference | **Punctate** | **Amorphous** | **Coarse heterogeneous** | **(Fine) pleomorphic** | **(Fine) linear (branching)** | **Other** |
| Avdan Aslan 2021 (16) | - | Amorphous | Coarse heterogeneous | Fine pleomorphic | Fine linear branching | - |
| Bae 2013 (17) | Punctate | Amorphous | Coarse heterogeneous | Fine pleomorphic | (Fine) linear branching | - |
| Bagnall 2001 (18) | Punctate | - | - | - | Linear | Granular |
| Barreau 2005 (19) | - | Amorphous | - | Fine pleomorphic | Fine linear branching | Milk of calcium/ round |
| De Roos 2006 (21) | - | - | Coarse granular | Fine granular | Linear | - |
| De Roos 2004 (20) | - | - | Coarse granular | Fine granular | Linear | - |
| Dinkel 2000 (22) | - | - | Coarse granular | Fine granular | Linear | - |
| Evans 2010 (23) | Punctate (round/oval) | - | - | - | Casting/linear | Granular/irregular |
| Hofvind 2011 (24) | Punctate | Amorphous | Coarse heterogeneous | Fine pleomorphic | Fine linear branching | - |
| Holmberg 2013 (25) | - | Powdery | - | Crushed stone-like | Casting-type | - |
| Kessar 2002 (26) | Punctate | - | - | Pleomorphic | Fine linear branching | - |
| Kim 2015 (27) | Punctate | Amorphous | Coarse heterogeneous | Fine pleomorphic | (Fine) linear branching | - |
| Kong 2020 (28) | - | Amorphous | Coarse heterogeneous | Fine pleomorphic | Fine linear branching | - |
| Lee 2000 (29) | Punctate | Amorphous | - | Pleomorphic | Fine branching | - |
| Lee 2021 (30) | Punctate | Amorphous | Coarse heterogeneous | Fine pleomorphic | Fine linear branching | - |
| Lilleborge 2021 (31) | Round/punctate | Amorphous | Coarse heterogeneous | Fine pleomorphic | Fine linear (branching) | - |
| Nishimura 2004 (33) | Punctate, Round | Amorphous | - | Pleomorphic | Linear, Branching | - |
| Rauch 2016 (34) | Punctate | Amorphous | Coarse heterogeneous | Fine pleomorphic | Fine linear branching | - |
| Rominger 2015 (35) | Punctate | Amorphous | Coarse heterogeneous | Fine pleomorphic | Fine linear branching/linear branching | - |
| Stomper 2003 (37) | - | - | Granular coarse | Granular fine | Linear | Granular mixed |
| Szynglarewicz 2016 (38) | - | Powdery | - | Crushed stone-like | Casting-type | - |
| Tabar 2011 (40) | - | Powdery | - | Crushed stone-like | Casting-type | - |
| Tan 2000 (41) | - | Powdery | - | Crushed stone-like | Casting-type | - |
| Thurfjell 2002 (43) | - | Amorphous | - | Pleomorphic | Fine linear (branching) | - |
| Wang 2019 (44) | - | Amorphous | Coarse heterogeneous | Fine pleomorphic | - | - |
| Woodard 2019 (45) | - | Amorphous | Coarse heterogeneous | Fine pleomorphic | (Fine) linear branching | - |
| Zhang 2021 (46) | - | Amorphous | Coarse heterogeneous | Fine pleomorphic | fine linear (branching) | Typical benign |
| Zhou 2017 (47) | - | Powdery | - | Crushed stone-like | Casting-type | - |
| Zhou 2014 (48) | - | - | - | Crushed stone-like | Casting type | All other calcifications |
